# FLIIMP - a community software for the processing, calibration, and reporting of liquid water isotope measurements on cavity-ring down spectrometers

**DOI:** 10.1016/j.mex.2023.102297

**Published:** 2023-07-27

**Authors:** Harald Sodemann, Pål Tore Mørkved, Sonja Wahl

**Affiliations:** a Geophysical Institute, University of Bergen, Bergen, Norway; b Bjerknes Centre for Climate Research, Bergen, Norway; c Department of Earth Sciences, University of Bergen, Bergen, Norway; d Alfred-Wegener-Institute Helmholtz Centre for Polar and Marine Research, Bremerhaven, Germany; e CRYOS, School of Architecture, Civil and Environmental Engineering, EPFL, Sion, Switzerland

**Keywords:** Stable water isotopes, Liquid water sample analysis, Cavity ring-down spectroscopy, Memory correction, Calibration, Long-term reproducibility, Post-processing and calibration of stable water isotope composition in liquid samples

## Abstract

Precise and accurate measurements of the stable isotope composition from precipitation, land ice, runoff, and oceans provide critical information on Earth's water cycle. The analysis, post-processing, and calibration of raw analytical signals from laser spectrometers during sample analysis involves a number of critical procedures to counteract instrumental drift, inter-sample memory effects, and the quantification of total uncertainty. We present a new software tool for the post-processing and calibration named FLIIMP (FARLAB Liquid Water Isotope Measurement Processor). FLIIMP facilitates sample processing by (1) a graphical user interface that guides the user along the processing steps from corrections for memory effects, drift, and mixing ratio to calibration, and (2) allows to monitor long-term measurement system behaviour, currently for Picarro-brand water isotope analysers. Final data files are accompanied by a detailed calibration report. Being an open-source software for the major operating systems, users can adapt FLIIMP to their laboratory environment, and the community can contribute the software development.

• FLIIMP facilitates post-processing, calibration and reporting for stable water isotope liquid sample analysis.
• The stepwise, interactive graphical user interface reduces possibility of errors and shortens processing time.
• Open source software enables future development of FLIIMP by the user community.

## Graphical abstract

**Figure:**
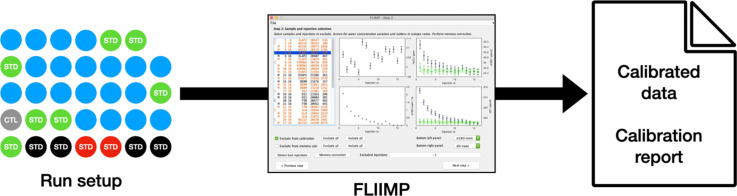

Specifications table

**Table:** 

Subject area:	Earth and Planetary Sciences
More specific subject area:	Environmental Sciences
Name of your method:	Post-processing and calibration of stable water isotope composition in liquid samples
Name and reference of original method:	Gröning, M., 2011. Improved water δ^2^H and δ^18^O calibration and calculation of measurement uncertainty using a simple software tool. Rapid Commun. Mass Spectrom. 25, 2711–2720, 10.1002/rcm.5074.
Resource availability:	The FLIIMP software is available in V2.0 for download as MATLAB source code or in compiled versions for Windows, MacOS and Linux from a public git repository at https://git.app.uib.no/GFI-public/fliimp. Installation instructions are available at https://git.app.uib.no/GFI-public/fliimp/-/wikis/Installation.

**Method details**

## Overview

Water isotopes are a valuable observed tracers in hydrology, hydrometeorology, and climate system studies (e.g., [2,^6^,8]). Highest analytical standards and consistent processing between laboratories are critical to enable the reliable use of published results, for example in meta-analyses with wider geographic coverage [4]. The recent advent of laser spectroscopy (e.g., [5]) has made the analysis of the stable isotope composition of liquid water samples more common, also in smaller laboratories. The post-processing and calibration (or normalisation) of the raw analytical signal of laser spectrometers used in liquid sample analysis needs to correct for instrumental drift and inter-sample memory effects [9,13], and should quantify the combined uncertainty [14]. Several solutions are available for the post-processing and calibration of samples according to recommendations of the Isotope Hydrology Section of the International Atomic Energy Agency (IAEA). SICalib is a widely used software application running in the Microsoft Excel spreadsheet software, covering all necessary processing steps for different analysers, including memory and drift correction, to calibration/normalisation, and provides complete uncertainty calculations ([10], 2018). LIMS for Lasers [7] covers all steps from customer details to sample handling, calibration, and system monitoring, and stores samples in a database. Both systems are only available for Windows operating systems.

Here we present the new software tool FLIIMP (FARLAB (Facility for advanced isotopic research and monitoring of weather, climate and biogeochemical cycling) LIquid Water Isotope Measurement Processor) for supporting the workflow of liquid sample analysis for water isotope composition. FLIIMP thereby adopts the recommendations of IAEA, and extends upon the functionality provided in SICalib and LIMS for Lasers by providing a streamlined graphical user interface (GUI), semi-automated inter-sample memory correction, the creation of a shareable calibration report, and support a greater range of computer operating systems. The GUI is set up in a logical sequence, and visually supports the processing during review of injections, corrections for memory effects and mixing ratio dependency, for drift, calibration and uncertainty assessment, thereby reducing chances to make (undetected) operator errors. In its current version 2.0, FLIIMP reads Picarro-brand water isotope analyser output files, and runs on Windows, MacOS, and Linux operating systems. FLIIMP is written in MATLAB, and can either be run from the MATLAB programming environment, or as a stand-alone compiled application. Published under a GNU Lesser General Public License Version 3 from an openly accessible git repository, FLIIMP can be modified and expanded in the future according to the needs of the user community. Here we describe the FLIIMP structure, capabilities and processing steps, proceeding along the sequence from data input, pre-processing, memory correction, to calibration, using examples from FARLAB. In the Appendix A, we provide a step-by-step overview of the typical sequence of operation, and the steps needed to adapt FLIIMP to a new laboratory environment.

## Installation and software structure

FLIIMP consists of a set of functions written in MATLAB available from a public git repository.^1^ The software can either be run with a MATLAB installation in version 2017 or later (license required), or as compiled software available from the git repository using a free MATLAB runtime environment installation. FLIIMP is installed by cloning the repository to a local computer, or by downloading the routines as a zip file. FLIIMP is then started by running the main routine FLIIMP.m within the source directory. More detailed installation instructions, including the steps to open a sample data file provided with the software, and for installing the compiled application, are available on the git repository's wiki pages.

**Fig. 1:**
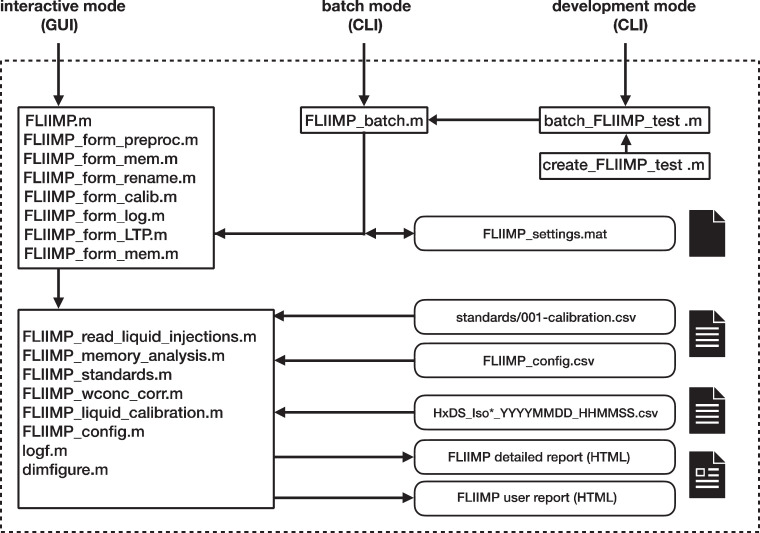
Structure of the FLIIMP software and names of program files for operation in interactive mode with a graphical user interface (GUI) or in batch mode with a command line interface (CLI). Input and output files are shown with a rounded shape.

In order to facilitate understanding of the processing steps, but also user configuration and potential modifications of the software, we briefly describe the general structure of the program code. The GUI is contained in the set of routines starting with FLIIMP_form_*.m, for example FLIIMP_form_preproc.m and FLIIMP.m (Fig. 1, top left box). More specific functionality is contained in separate routines, such as for file input (FLIIMP_read_liquid_injections.m), memory correction (FLIIMP_memory_analysis.m), mixing ratio correction (FLIIMP_wconc_corr.m), and calibration, reporting and data file output (FLIIMP_liquid_calibration.m). Configuration for a new laboratory can to a large extent be done with modification of MATLAB code by changing parameters in the comma separated values (csv) formatted text file FLIIMP_config.csv, located in the installation folder (Appendix B). The entire program code of FLIIMP is extensively documented to facilitate modifications by others.

During processing, FLIIMP reads and writes several types of files (Fig. 1, round boxes). Regarding input files, FLIIMP is in the current version only able to read csv-formatted data files from Picarro-brand L2130-i, L2140-i, and many older analysers (Picarro Inc., Sunnyvale, USA). In the future, we may add functionality to read output files from other analyser brands. FLIIMP automatically detects data files that contain measurements of δ^17^O, and in that case displays corresponding visualisation and processing options (Sec. 3.7). Importantly, FLIIMP does not require a specific run setup in terms of standard and sample vial arrangement and number of injections for processing. This flexibility facilitates the testing and optimisation of different run setups for different applications, which we consider an improvement over existing solutions. While we in this manuscript use examples based on the FARLAB run setup (Appendix A.1), we emphasise that users can operate FLIIMP seamlessly with entirely different run setups.

All processing steps in FLIIMP, including for example the re-naming of mis-labelled samples, are performed on top of the original data files. There is thus no need to maintain a database application or to make copies of analyser output files. Original water isotope analyser output files can for example be archived in read-only mode at a central network attached storage, while FLIIMP run settings only contain the operation steps to be performed on these data files, and are archived with the processing reports and calibrated data at a different storage location.

FLIIMP can either input find data files automatically in a default file structure when instrument and measurement start time are specified, or users can manually select input files. To automatically locate files, data files are expected to be located in a common directory structure that defaults to <base path>/Instruments/<Instrument name>/IsotopeData/. The naming of input files should then comply with a format that defaults to <device>_IsoWater_<date>_<time>.csv (Sec. 3.1). The path structure and naming format of run input files can be modified in configuration file FLIIMP_settings.csv (Appendix B).

Output data files are created in csv format (Sec. 3.6), and accompanied by a detailed illustrated report in hypertext markup language (HTML) for sharing with users, or with additional details for laboratory-internal use (Fig. 1, bottom right). The uniform HTML reporting in FLIIMP allows the operator to inform customers in detail about the processing of their samples, including calibrated values, uncertainty, the processing methods, and recommended acknowledgements. Text blocks to be included in the report can be specified in the configuration file FLIIMP_config.csv. Some of the FLIIMP settings, including the last choice of analyser, input and output path, and window locations, are saved in the user's home directory in file FLIIMP_settings.mat for re-use in the next FLIIMP session.

FLIIMP can be operated either with mouse and keyboard through a GUI (Sec. 3.1), or from the command line in so-called batch mode (Sec. 4.3). Both processing modes give the same result, except that the batch mode does not allow for interactive manipulation of parameters, and requires a MATLAB installation. Commonly, users will mainly operate FLIIMP in interactive mode with the GUI. It is recommended to save the processing setup for traceability and later re-use for each run, for example for re-processing data files from older runs in batch mode if updated correction methods become available.

## Data processing

### Data file import (Step 1)

**Fig. 2:**
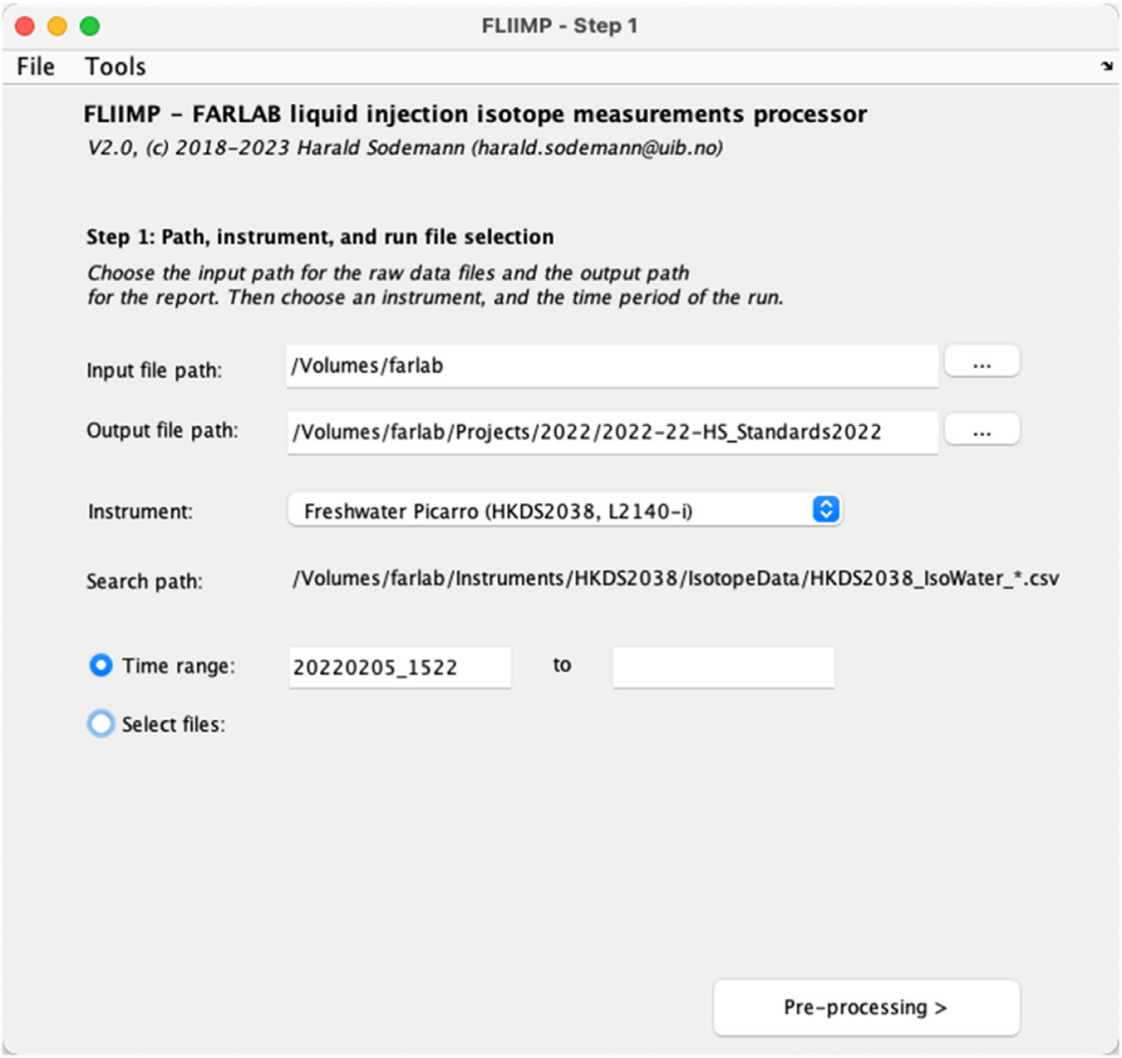
Start panel of FLIIMP with GUI elements to specify the input file, analyser, and file locations.

FLIIMP in interactive mode is started by running the main routine FLIIMP.m in MATLAB or the icon of the compiled app. After start, FLIIMP presents a window with the first processing panel (Step 1, Fig. 2). Here, the user enters input and output paths, select the instrument on which the run was performed, as well as the time range of the analysis or make a manual file selection (Fig. 2). Based on the user entries, FLIIMP displays the current search path for instrument file input above the date entry fields. If a run consists of several input files, both the start and end date have to be specified (format YYYYMMDD_HHMMSS, or parts thereof). The entered date will be used as part of a pattern matching to find all relevant files (e.g., 20220501_01*). If a run is contained within exactly one output file, no end date needs to be specified. If users prefer, and in the case that file name does not match the expected pattern (Appendix B.2), for example when using manually edited files, it is also possible to directly select and add the files to be processed by using the option *Select files*. Users can test FLIIMP with an input data file provided as part of the git repository^2^ and described in the installation notes.

### Pre-processing procedures (Step 2)

**Fig. 3:**
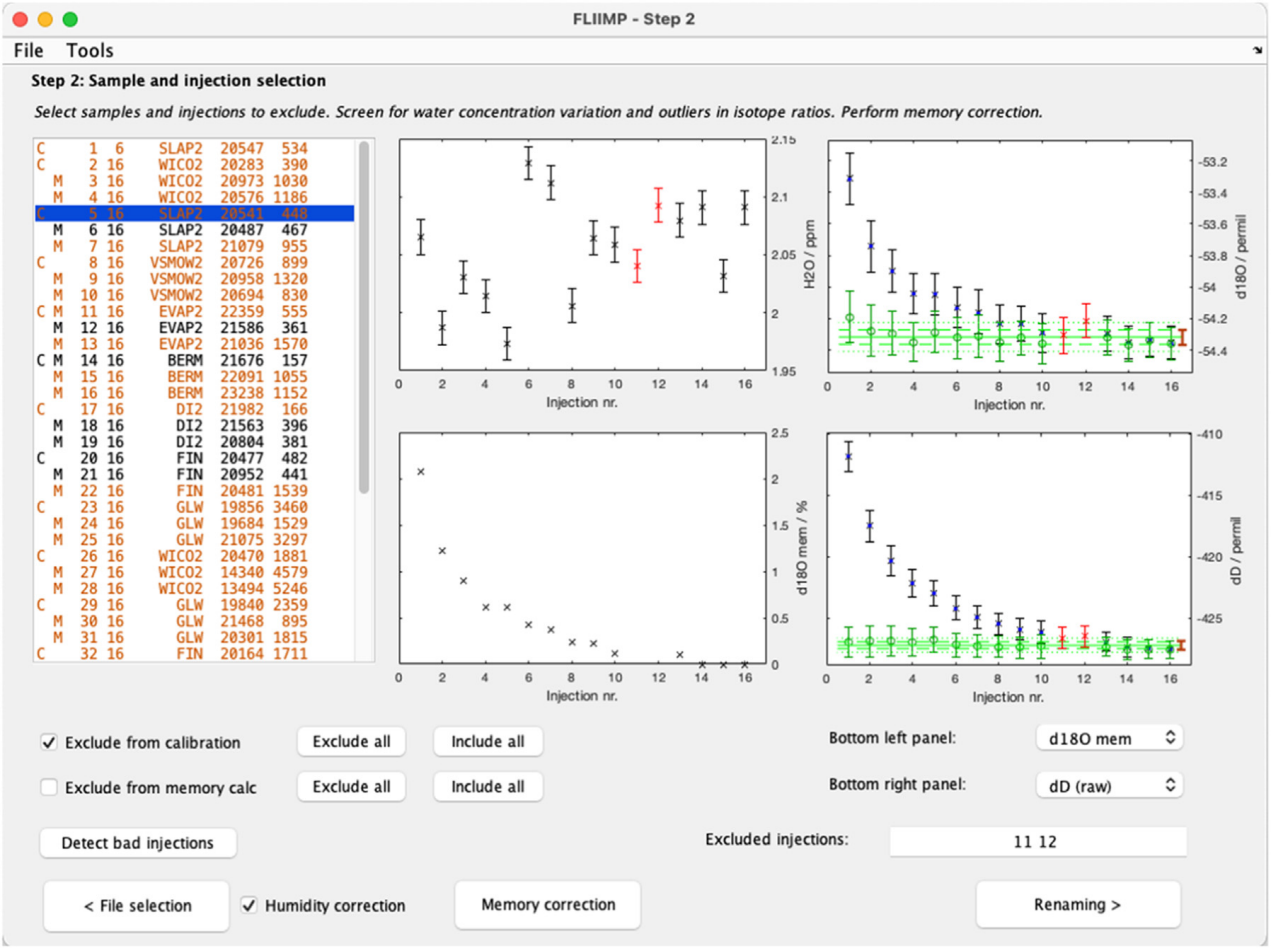
User interface for Step 2 (sample pre-processing) in FLIIMP. The green symbols in the panels on the right-hand side indicate the result of memory correction applied to the raw delta values (black and blue markers). Orange text in the left panel indicate samples that either have large inter-sample mixing ratio variations, or large standard deviations of the mixing ratio during one injection.

We now return to processing in interactive mode. At the start of Step 2, the software tries to load and combine all input files that match the specified instrument and time patterns specified in Step 1, and presents these in the window for sample screening (Fig. 3). FLIIMP allows the user to perform four pre-processing steps before calibration that can correct for or help identify typical analytical problems during a run: (i) sample and injection outlier screening, (ii) memory correction, (iii) correction of mixing-ratio dependent baseline effects, and (iv) assignment of quality flags.

#### Sample screening

Liquid injection measurements involve several mechanical steps, that can cause variations in measurements [9]. A common consequence is that some (or all) injections of a sample fail, for example due to syringe wear and clogging. Some samples also need to be excluded for the final reporting, for example in case of duplicates (see Appendix A.1). Other situations that may require sample exclusion are partially completed runs, or pre-conditioning samples for memory correction (see Sec. 3.3).

After clicking on the *Read files* button, the data files are loaded into FLIIMP, and the software proceeds to Step 2. Any error messages, for example if no files could be found, are displayed in the FLIIMP log window. At any processing step, the current processing settings for a run can be saved or loaded in *.mat format using the *Save* or *Load* menu items from the *File* menu at the top of each window. It is recommended to save the run settings for every run or batch on the analytical system for traceability and to enable reprocessing at a later time. The structure and content of the settings files is detailed in Appendix B.4.

In FLIIMP, individual injections or entire samples can be excluded from further processing from the GUI. In the interface for sample pre-processing (Fig. 3), a table on the left side displays the sample names and IDs, along with the number of injections, and the standard deviation of mixing ratio. Orange colour denotes samples that should be inspected in detail due to one of several potential error sources, such as large standard deviation in mixing ratio or isotope δ-value. Clicking on a sample name will display a corresponding error bar graph in the panels to the right. In addition to the mixing ratio and δD, the pull-down menus allow to plot also the δ^18^O, δ^17^O, d-excess, ^17^O-excess in ‰, as well as the memory effect in% once it has been quantified (Sec. 3.3). Operators are advised to inspect each sample of a run.

Entire samples are ignored from further processing by selecting a sample in the sample list, and activating the *Exclude from calibration* checkbox. If a sample should only be excluded from memory correction, the *Exclude from memory calc* checkbox can be used. Correspondingly, samples can be included in the memory correction for their large difference in δ-value between sequential samples, while being ignored from calibration, since it served as a duplicate during the calibration sequence.

Individual injections from within a sample can be marked as outliers by clicking below or above the error bar symbols in one of the display panels, or by typing the corresponding injection number in the text field below the preview of the selected sample. Excluded injections will be shown in red colour in the panels. Outliers are not taken into account for the maximum and minimum vertical range of the plots, and therefore not always displayed entirely. Operators are advised to only exclude samples when justified. We have found that on some analysers the data acquisition rate sporadically drops to substantially less than the usual about 0.75 Hz of Picarro's L2140-i. The injections during these cases are distorted, and need to be excluded from further analysis. Pressing the button *Exclude bad injections* will identify injections with frequencies lower than ∼0.65 Hz, and add them to the list of outliers. Excluded samples and injections are listed in the detailed calibration report in report section 8 (Sec. 4).

#### Mixing ratio-isotope ratio dependency correction

Humidity variations between injections are a common measurement artifact of liquid water analyses. According to specifications, Picarro analysers perform optimally for a mixing ratio of between 17′000 to 23′000 ppm [9,17]. In this range, spectroscopic baseline effects due to variable humidity are nearly constant. If the mixing ratio falls outside a range of about 15′000 to 25′000 ppm, injections either need to be discarded, or one can apply a correction method. It has long been documented that the baseline effect of these analysers is instrument dependent, but can be characterised by careful laboratory analysis, and thus be corrected for (e.g., [1,^3^,18]). Weng et al. [19] showed that the artifact is both a function of the mixing ratio and the isotope ratio, and that it differs depending on the matrix gas of the analyser. If the dependency functions for mixing ratio and isotope ratio are known, for example from the procedures presented in Weng et al. [19], FLIIMP can automatically correct individual injections with lower or higher humidity. Corrected mixing ratio and isotope ratios are shown in magenta colour in the mixing ratio plot (Fig. 3, center top panel). The mixing ratio-isotope ratio correction assumes that the mixing ratio was constant during the measurement for a specific injection. In some cases, such as vaporiser septum leakage, the mixing ratio signal can show much larger slopes than the typical slope of about 200 ppm min^−1^ when sampling from the vaporiser, causing a larger standard deviation for that injection. Such injections cannot be corrected properly, and should be excluded manually based on their inflated error bars in the mixing ratio display. If the correction functions are not yet determined for the used analysers, samples with low or high mixing ratio can be excluded as outliers (see Sec. 3.2.1).

Even though the correction functions for vapour analysis are non-linear functions, it is possible to approximate these with linear relations within the range most common for liquid injections analysis (typically about 10′000 to 30′000 ppm). In the routine FLIIMP_wconc_corr.m, the correction functions f (x) are specified for each individual analyzer in a laboratory, and for each isotope species, in the form

(1) $$\begin{array}{c} f\left(x\right)=\delta_{\mathrm{corr}}\left(x\right)-\delta_{\mathrm{raw}}\left(x\right)=\mathrm{ax}+b \end{array}$$

Thus, the corrected δ_corr_ results from an offset of the raw δ_raw_ by a quantity obtained from a linear fit of raw mixing ratio x to the corresponding δ-value correction f (x) with slope a and intercept b. Alternatively, a wider range of corrections can be achieved with hyperbolic fitting functions of the following form:

(2) $$f\left(x\right)=\frac{1}{\tilde{x}}a+b\tilde{x}+c.$$

Hereby, a, b, and c are fitting coefficients that depend on isotope ratio, and $\tilde{x}=x-x_{\text{ref}}$, and $x_{\text{ref}}=\;20{}^{\prime }000$ ppmv. Note that as mentioned above, these correction functions are in addition to the delta value dependent on the matrix gas, and are specific to each analyser [19]. Several other correction functions have been used in the literature previously (e.g., [9,14]) which could be implemented as alternative options in routine FLIIMP_wconc_corr.m.

#### Quality flags and quality control

In order to enable a correct interpretation of the calibrated measurement results, all available information about analytical data quality, such as measurement artifacts, and the applied correction, need to be documented clearly. Therefore, quality flags are assigned to each sample that signify information and warnings about the run, and about the applied post-processing steps. Operators of FLIIMP should check for combinations of several flags, which often indicate problems during the analytical procedure, such as a worn-out syringe. All flags are written out as a single-value bit combination in the calibrated data file (Table 1), and detailed further in the data report. For example, a sample where the mean of the standard deviations from each individual injection exceeds 200 ppmv would receive flag value 1, which can indicate problems with the peak shape. A sample where the mean of the standard deviations of the mixing ratio of all considered injections is larger than 500 ppmv would receive a flag value 2, potentially indicating problems with the syringe or septum. A sample where in addition the standard deviation of instrument temperature exceeded 0.15 K would receive the quality flag value 2+16=18. Measurements with no issues at all have a flag value of 0.

**Table 1:** Quality flags for sample analysis, assigned during the pre-processing with FLIIMP.

Flag nr.	Description	Value	Criterion
1	Humidity variation during injections	1	mean(SD(w))> 200 ppmv for averaged injections
2	Humidity variation between injections	2	SD(w)>500 ppmv for averaged injections
3	Large isotopic variability	4	SD(delta_D)>0.5 ‰ or SD (delta_18O)>0.15 ‰
4	Sample outside range of standards	8	delta_D or delta_18O of sample larger or smaller than calibration standards
5	Instrument temperature variability	16	SD(T_DAS)>0.15 K
6	Picarro error codes set	32	not used

### Memory correction (Step 3)

**Fig. 4:**
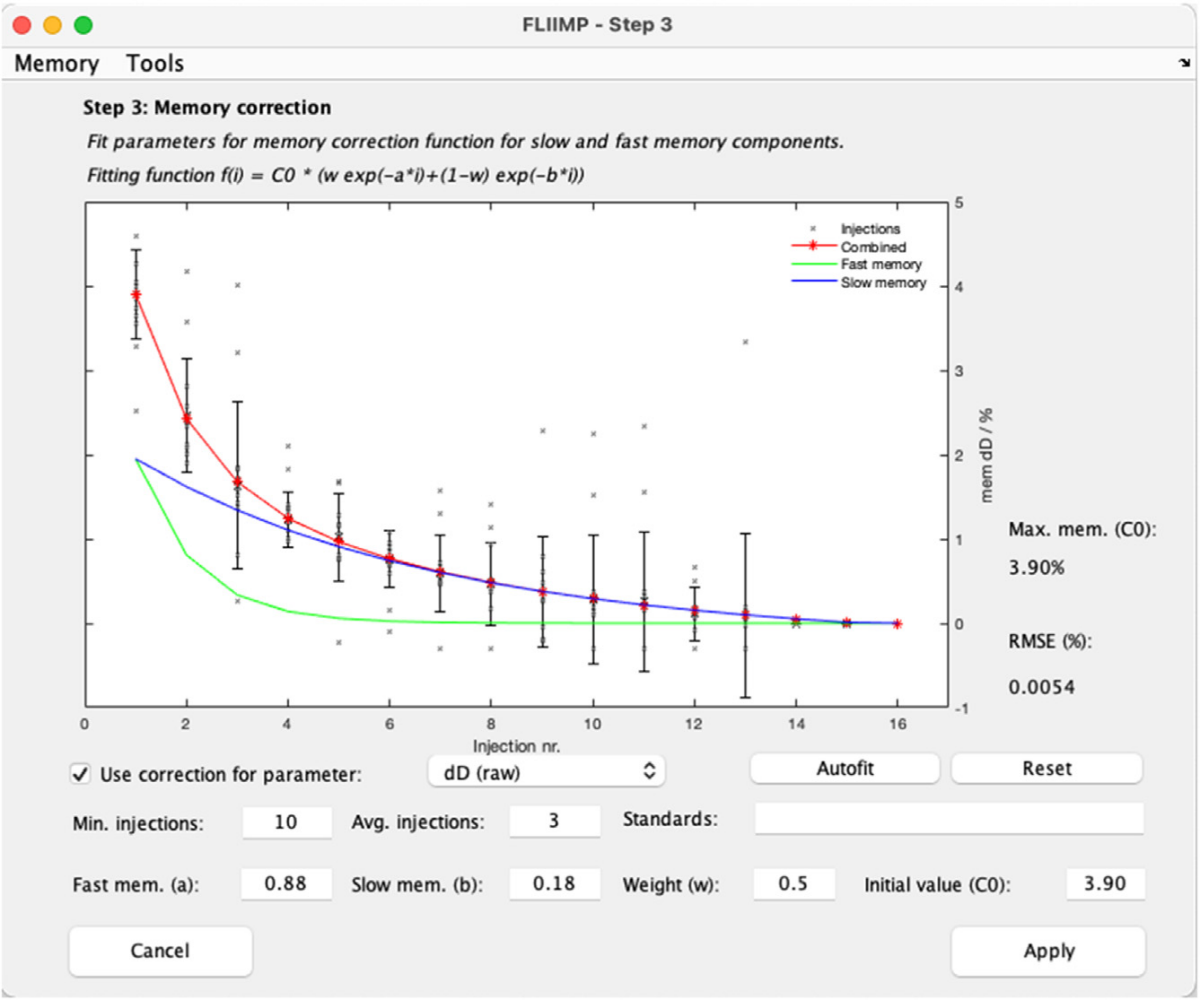
Memory correction in FLIIMP. Computation of memory correction for δD based on all samples that have 10 injections or more. In the presented example, laboratory standards are measured with 10 injections, and samples with 6, such that the memory correction is only based on standards.

Step 3 in the FLIIMP processing sequence consists of the user interface to correct for inter-sample memory effects (Fig. 4). FLIIMP memory correction uses a two-component model, and assists the user by automatically obtaining the optimal fitting parameters. This step is available from the button *Memory correction* in the pre-processing window (Fig. 3). First, the underlying correction algorithm is described, before describing the corresponding GUI.

#### Memory correction method

An important measurement artifact during laser spectroscopy of liquid samples are memory effects. Memory effects result from the carry-over of sample material on walls and other parts of the analyser between samples. Several methods have been proposed in the past to reduce memory effects between injections, and to correct for memory effects as part of post-processing [9]. FLIIMP has the option to correct all samples of a run based on memory correction coefficients obtained typically from a set of injections of calibration or drift standards, that have a sufficiently large number of injections. Similar to Gröning [10], the memory correction in FLIIMP is based on a model that combines the effect of a slow and a fast memory component for each injection j:

(3) $$M_{j}=C_{0}\;\left(we^{-\text{aj}}+\left(1-w\right)e^{-\text{bj}}\right)$$

The two memory components contribute to memory as described by exponent a and b for the fast and slow processes, respectively, that decreases to 0 at *N*−n injections, where N is the total number of injections. The total memory M_j_ is then obtained by a weighted mean of both memory components, using the weight factor w where w ∈ [0 . . . 1] (Eq. (3)), multiplied with the maximum, initial memory C_0_ for injection *j*=1. Thereby, C_0_ is obtained in a three-step process. First, the final value of the raw δ-value, ${\tilde{y}}^{i}$, is calculated for each sample y^i^, using the last n of N injections:

(4) $${\tilde{y}}^{i}=\langle y_{N-n}^{i},...,y_{N}^{i}\rangle$$

Then, the difference between the final δ-values of consecutive sample *i*−1 and i is used to compute the percent memory M^i^_j_ affecting each sample i at injection j:

(5) $$M_{j}^{i}=\frac{{\tilde{y}}^{i}-{\tilde{y}}^{i}}{{\tilde{y}}^{i-1}-{\tilde{y}}^{i}}\cdot 100\%$$

Note that negative values are not excluded during memory correction to prevent a positive bias when fitting the memory correction.

When sequential samples have small difference in isotope ratio, the memory quantification becomes more uncertain. Therefore, all available memory estimates are weighted by their relative contribution to the sum of the absolute value of the step between final δ -values from consecutive samples *i*−1 and i:

(6) $$\beta^{i}=\frac{\left|{\tilde{y}}^{i-1}-{\tilde{y}}^{i}\right|}{\sum \left|{\tilde{y}}^{i-1}-{\tilde{y}}^{i}\right|}$$

Weighting of each memory estimate for each injection by β^i^ gives more weight to memory estimates that have been obtained from a large inter-sample difference of δ-values. Based on the weighted individual memory estimates for each sample and injection, $M_{j}^{i}\cdot \beta^{i}$, the memory model (Eq. (3)) can then be fitted, using root mean square error (RMSE) minimisation. Averaging over a larger number of injections n lowers sensitivity of the correction to other sources of variability, while requiring sufficiently many injections.

After fitting, the computed memory correction ${\tilde{M}}_{j}^{i}$ for each sample i at injection j ∈ [2 … N] is forced to zero at the last n values using a linear deprecation of the form

(7) $${\hat{M}}_{j}^{i}={\tilde{M}}_{j}^{i}-{\tilde{M}}_{N-1}^{i}\cdot \left(1-\left(N-j\right)/N\right).$$

FLIIMP then applies the computed memory correction ${\hat{M}}_{j}^{i}$, resulting from the deprecated superposition of the two exponential correction functions to all samples. In the program code, all memory processing is done in routine FLIIMP_memory_analysis.m.

#### Applying memory correction from the GUI

Memory correction is started in the GUI by pressing the button Memory correction in the pre-processing panel (Fig. 4). One can either choose to select the samples for the memory correction based on the number of injections (e.g., 12 for standards, compared to 6 for samples), or from the sample name identifying a standard. Several standards can be specified when separated by blank characters in the field Standards. FLIIMP displays black cross markers for the memory from all individual injections that match the selection criteria. In addition, and for the fitting, error bar markers show the weighted average of the memory estimates for each injection.

Pressing the *Autofit* button will run through a combination of parameters a, b, and w, to find the best fit from minimising the RMSE. When autofitting is done, the lines for slow memory (blue), fast memory (green), and total (red) will be updated in the display, as well as the numbers in the interface. Next to the display, the initial memory and RMSE value are displayed. If needed, it is possible to adjust the results of the autofitting manually. The display will update after pressing the enter key when editing fitting parameters. The procedure needs to be done for each isotope species (δD, δ^18^O, and δ^17^O, if applicable). Importantly, users need to check the box *Use correction for parameter* if they wish to apply the memory correction for each isotope species. Once a memory correction has been established, and the interface window is closed, the memory corrected values are shown in green colour in the panels on the right in the pre-processing window (Fig. 3).

In some situations, for example when measuring samples within a narrow range of isotope ratios, the memory correction is either inefficient, or can even degrade the calibrated results. Therefore, memory correction can be deactivated for each isotope species in the memory correction panel, or altogether in the run settings panel (Sec. 3.5.2). To decide whether to activate memory correction or not, it is important to review the quality of the correction for each sample after closing the memory correction panel. Operators are advised to check for all standards and samples that the memory correction has reduced the memory effect sufficiently, as evidenced by the green markers being nearly flat, and their 1-σ standard deviation (Fig. 3, green dashed lines) near the long-term reproducibility (Fig. 3, brown error bar).

In the FLIIMP report, a figure similar to Fig. 4 is displayed for all species in the detailed calibration report section 5. In addition, the memory correction is documented in the form of the initial memory C_0_, as well as the exponents a, b for all species. Both quantities are also included in the parameter data file for performance monitoring (see Sec. 4). Initial memory for δD and δ^18^O can be used to monitor system performance over longer times. Increased memory may for example indicate salt buildup, or other contaminations, and help to identify when cleaning of the analytical system is needed [14]. The trend of the initial memory and several other run parameters can be inspected for a selected instrument and time period using the menu item *Tools, Parameter analysis* (not shown), implemented in routine FLIIMP_form_memdrift.m.

Note that memory correction only works between two sequential samples with a sufficiently large difference in δ-values. The threshold value for memory correction can be specified in the FLIIMP settings variable memory_limits, and is by default 12.0 ‰ for δD and 1.5 ‰ for δ^18^O and δ^17^O. Experience with the measurement system indicates, however, that memory effects also carry over between more than two sequential samples, even though to a lesser extent. Such memory effects can currently not be corrected by FLIIMP post-processing. If there are large steps in isotope ratio expected between samples, duplicating samples directly after one another is an efficient measure to minimise such a memory effect. The duplication of sample material is also used in the run setup at FARLAB (see Appendix A.1). In such cases, the duplicate samples are often excluded from calibration, but may be used for memory correction.

### Changing sample description and sample ID (Step 4)

Step 4 in the FLIIMP processing sequence is a user interface to change sample names and sample identifiers (IDs). The distinction between sample names and sample IDs allows to trace each sample with a unique identifier during analytical procedures, while keeping the customer's sample names intact. However, sometimes operators make mistakes in the naming of either sample names or identifiers, such as swapping the position of the two. To avoid manually changing the input csv files, FLIIMP allows to easily re-assign and swap sample names and sample IDs. Thereby, the name changes are applied as a (saved) operation during the sample processing, keeping the original data files without modification.

**Fig. 5:**
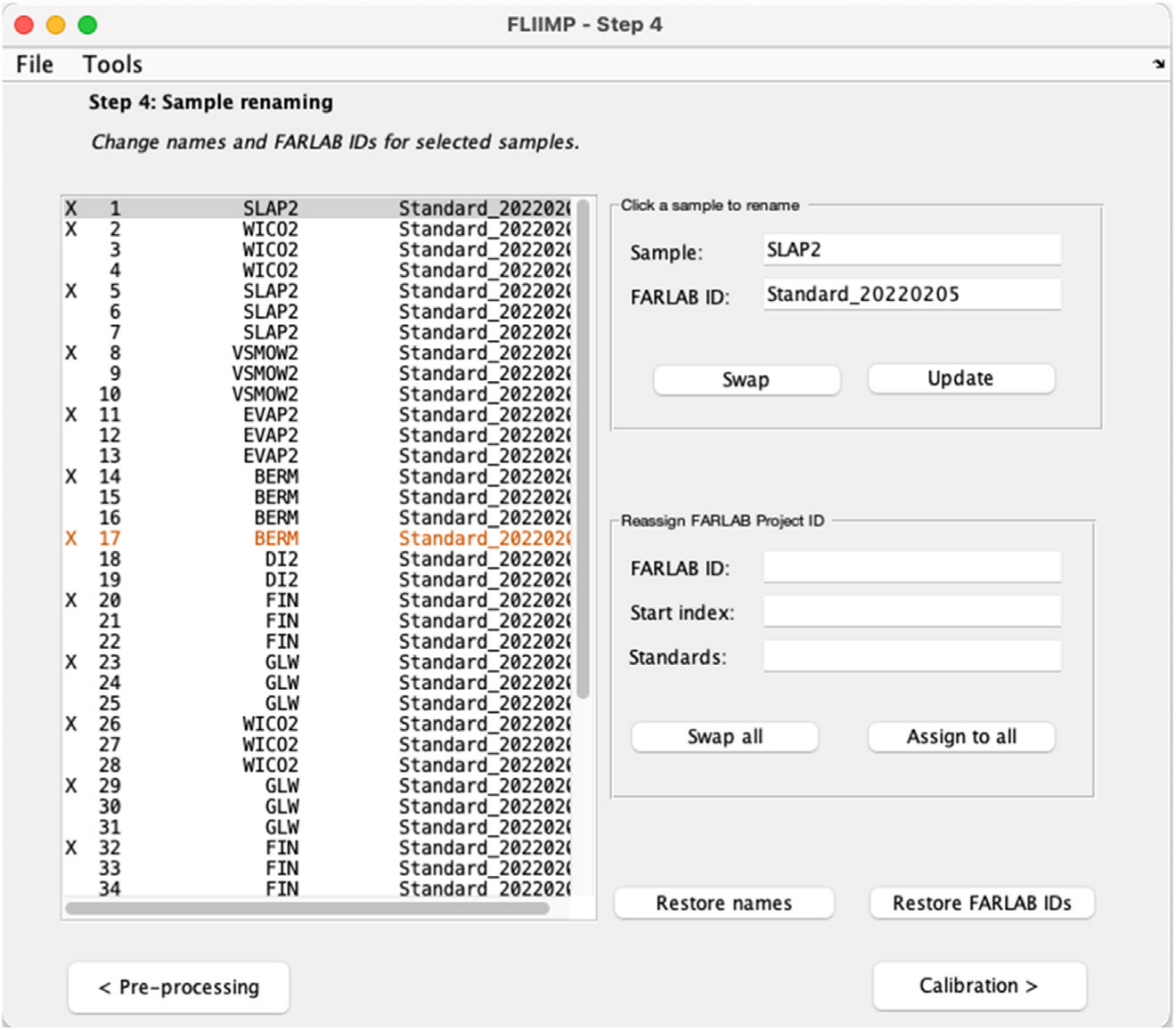
User interface for sample renaming and re-assignment of sample identifiers in FLIIMP (Step 4). See text for details.

To edit individual sample names and identifiers, users click on a line in the table on the left, and edit directly in the edit fields in the top right (Fig. 5). If all or several changes are desired, the lower right controls allow to swap all sample names and sample IDs, or to assign a new sample ID, based on a base string, and appending a numbered sequence starting at the value provided in *Start index*. Samples with names provided in the field *Standards* (space separated) are thereby skipped over. Both, original names, and original sample IDs can be restored any time during the renaming step. Modified table entries are highlighted in orange colour.

### Sample calibration/normalisation (Step 5)

Step 5 in the FLIIMP processing sequence is the specification of the information needed for producing the report, and for performing the calibration (or normalisation) to VSMOW-SLAP scale. First, the instrument drift during the run can be quantified and assessed. Furthermore, inspection of control standards allows to judge the performance of the measurement system during a given run. Finally, the processing report is exported, containing all information for internal monitoring or for an end user.

**Fig. 6:**
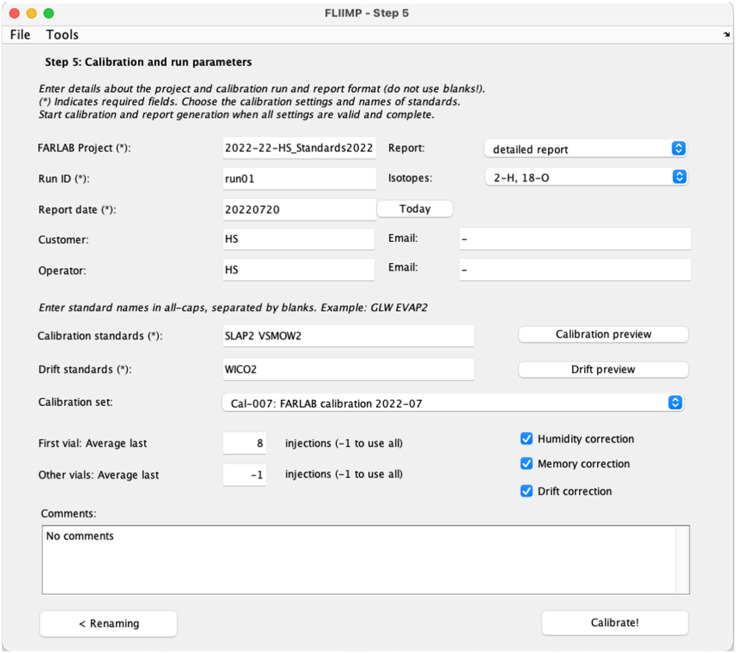
User interface for Step 5: report and calibration settings in FLIIMP.

All calibration parameters are specified in the GUI during step 5 (Fig. 6). These parameters include the calibration set, the calibration standards (with names separated by space), and the drift and control standards. It is also possible to activate or deactivate different pre-processing steps (humidity dependency correction, drift correction, and memory correction) during this step. In particular without memory correction, it may be desirable to restrict the processing to the last n injections of a sample. Specifying a value of −1 will use all available injections in the processing of each sample. Operators will also at least need to provide a project name and a run identifier. After completion of the specification, FLIIMP is ready for calibration of the samples, which is started by a click on the *Calibrate* button. FLIIMP will then create the output files in a new folder, including the calibration report (Sec. 4).

#### Drift correction

Drift correction is done before the actual calibration step. First, the slope of the drift is determined from a linear regression of the raw delta values of each of the drift standards against time. Then, the drift is shifted vertically, such that zero correction is applied at the middle of the run, since it is equally likely that the instrument drift is low at the beginning as at the end of the run, thus weighting the correction equally [10]. Instrumental drift is in particular relevant for runs that extend over several days. The drift correction is contingent on a suitable run setup. In an analytical system that can drift, the time used for measuring standards and for measuring samples needs to be optimised, to be able to detect and correct for drift, while measuring sufficiently many samples. A major complication thereby is the separation of instrument drift from memory effects, as well as other sources of variability. As described in the run setup (Appendix A.1), FARLAB uses duplicate samples of the drift standards to reduce the impact of memory effects on the drift correction. Therefore, one needs to exclude the first sample of the drift standard duplicates from calibration during sample pre-processing (Sec. 3.2.1).

**Fig. 7:**
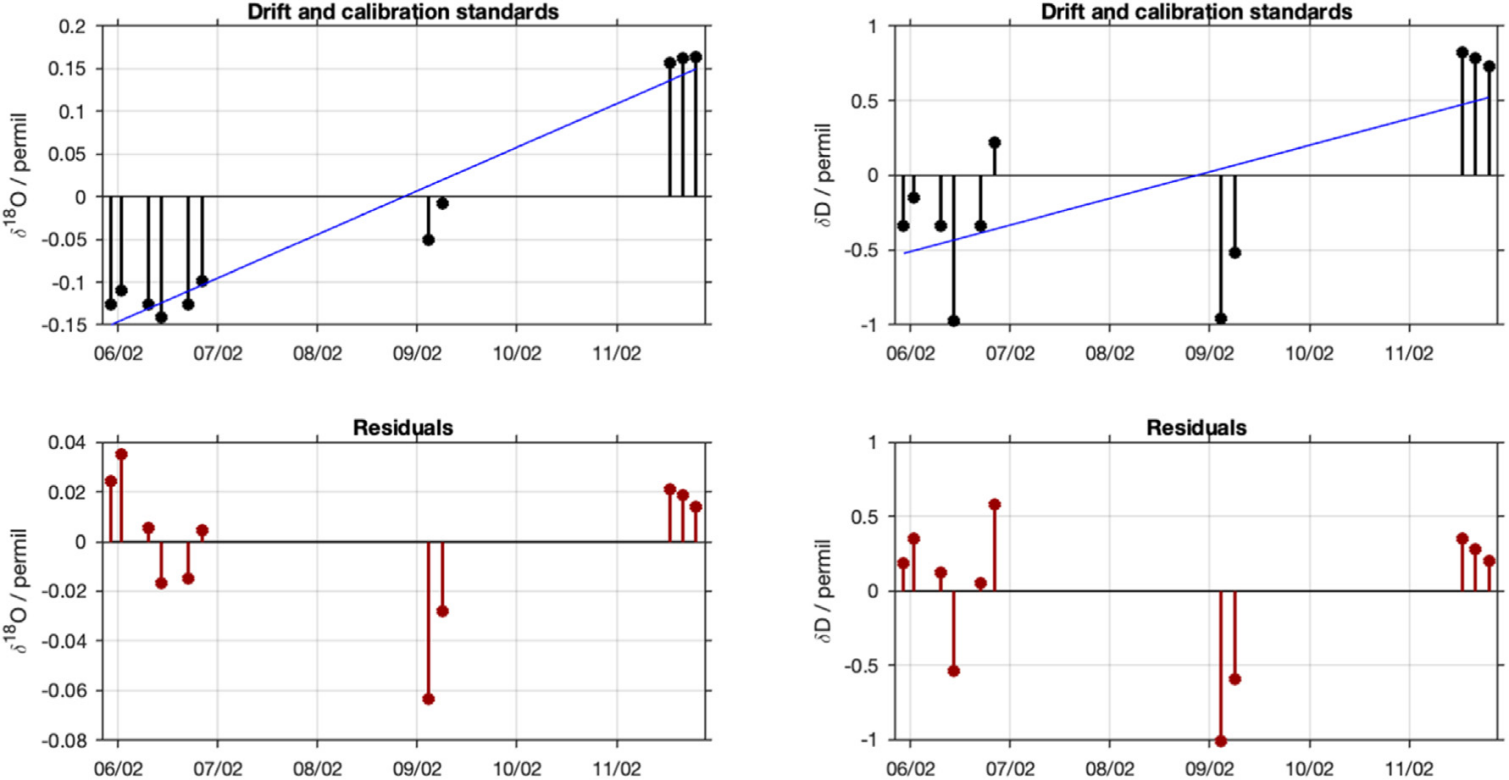
Example for the assessment of the drift and the quality of the drift correction during a run. (a) deviation of (a) δ^18^O (‰) and (b) δD (‰) from the average of each standard during the entire run with time. (c) Residuals after subtracting a linear drift relation for δ^18^O (‰) and (d) δD (‰) on the same time axis. Identical figures are included in the data report in report section 8.

With FLIIMP, laboratories can choose other suitable run setups to correct for drift. The drift correction is calculated in FLIIMP from the deviation of all available repeated standard measurements (drift, calibration, control) from their respective initial uncalibrated δ value. A linear fit to these offset values provides the mean drift of the measurement system in units of ‰ day^−1^ (Fig. 7a,b). The interpolated linear drift is then subtracted from all samples before calibration. The residuals from the linear drift estimate quantify the deviation from assumed linearity (Fig. 7c,d). From the GUI for step 5, the drift correction result figure can be previewed using the button *Drift preview* (Fig. 6). If desired, drift correction can be deactivated in the GUI during step 5 using the checkbox *Drift correction*.

#### Calibration to VSMOW-SLAP scale

Calibration (or normalisation) to VSMOW-SLAP scale is commonly done using secondary laboratory standards that have been calibrated against primary standards available from IAEA. The assigned values of secondary laboratory standards are provided as csv files in subfolder standards in the FLIIMP source code directory. As assigned values may change over time, or different waters may be added, new calibration sets can be added to that folder using a specific naming scheme (Appendix B.1). In addition to the assigned value, the combined uncertainty of the secondary (and primary) standards needs to be specified to allow for a correct calculation of the combined uncertainty of the calibrated samples. When reprocessing in batch mode, a different calibration sets can be selected using the settings parameter calset (Appendix B.4).

Calibration itself is done following recommended IAEA procedures [12]:

(8) $${\delta D}_{\text{smp}}^{c}={\delta D}_{\text{LS}1}^{c}+\left({\delta D}_{\text{smp}}^{w}-{\delta D}_{\text{LS}1}^{w}\right)\cdot f$$

(9) $$f=\left({\delta D}_{\text{LS}2}^{c}-{\delta D}_{\text{LS}1}^{c}\right)/\left({\delta D}_{\text{LS}2}^{w}-{\delta D}_{\text{LS}1}^{w}\right)$$

Hereby, ${\delta D}_{\text{smp}}^{c}$ is the calibrated δ value of the sample, ${\delta D}_{\text{LS}1}^{c}$ and ${\delta D}_{\text{LS}2}^{c}$ denote the calibrated values of working standards 1 and 2, and superscript w denotes the raw values of the standard and sample. Corresponding equations exist for the other isotope species.

The processing report contains a figure to control the quality of the calibration to VSMOW-SLAP scale. Samples are displayed at their position along the calibration line, which informs about the contribution from the calibration standard uncertainty to combined uncertainty. In addition, warnings are issued in the log window and report if samples are outside the standard range, and a corresponding warning flag is set (Sec. 3.2.3).

#### Uncertainty calculation

Combined uncertainty of the calibrated samples is estimated from an error budget, involving the following components ([10,14]):

1. u(h)^2^: variance from the assigned uncertainty of isotopically heavy standard h with respect to VSMOW-SLAP
2. u(l)^2^: variance from the assigned uncertainty of isotopically light standard l with respect to VSMOW-SLAP
3. u(H)^2^: variance from uncertainty expressed as standard error of the mean (SEM) of measured values of isotopically heavy standard H (SD for a single measurement)
4. u(L)^2^: variance from uncertainty expressed as SEM of measured values of isotopically light standard L (SD for a single measurement)
5. u(m)^2^: variance of sample (unknown). Approximated by repeated measurements or by long-term reproducibility and repeatability. If no value for the long-term reproducibility is specified (Table B.1), this value is estimated by the scaled SEM of each repeated sample measurement. The combined uncertainty u(c) is then calculated from the square root of the squared sum of all error components in the budget using
  (10) $$\begin{array}{c} u\left(c\right)=\sqrt{s_{h}^{2}u{\left(h\right)}^{2}+s_{l}^{2}u{\left(l\right)}^{2}+s_{H}^{2}u{\left(H\right)}^{2}+s_{L}^{2}u{\left(L\right)}^{2}+s_{m}^{2}u{\left(m\right)}^{2}} \end{array}$$

where s_h_, s_l_, s_H_, s_L_ and s_m_ are sensitivity terms of the form $s_{h}=\partial f/\partial \delta_{h}$, corresponding to each of the five elements of the error budget. Hereby, f represents the calibration function (Eq. (9)). Near the centre of the two-point calibration curve, samples obtain a lower uncertainty than near the edges [11]. Adding more standards to obtain a three-point calibration curve, for example, has been shown to be less important than obtaining precise and accurate measurements for a two-point calibration [16]. FLIIMP does therefore currently only offer a two-point calibration. The FLIIMP data report explains the processing steps and the calculation of combined uncertainty for each batch of samples in report section 4. The detailed report also contains a table specifying the full uncertainty budget of each sample.

An important consequence from the uncertainty budget is that calibration standards spanning a wider range will improve the uncertainty of calibrated samples located in between [16]. However, using calibration standards with very different isotope composition introduces more memory, and requires either more injections or other methods to obtain an accurate calibration. In the currently used setup for runs at FARLAB, for example, all standard vials are duplicated or triplicated (Appendix A.1). The first vial of a standard allows to quantify memory, but is ignored during the calibration. The second and third vial of the same standard are then used during calibration only. In the case of far different δ-values of the calibration standards, the final value of the second vial can be up to 1 ‰ different in δD after 12 injections due to inter-vial memory. In the future, it may be possible to attempt correcting for inter-vial memory effects in FLIIMP. We emphasise here again that users may choose different run setups to reduce the combined uncertainty, which FLIIMP will be able to handle seamlessly.

#### Control standards

**Fig. 8:**
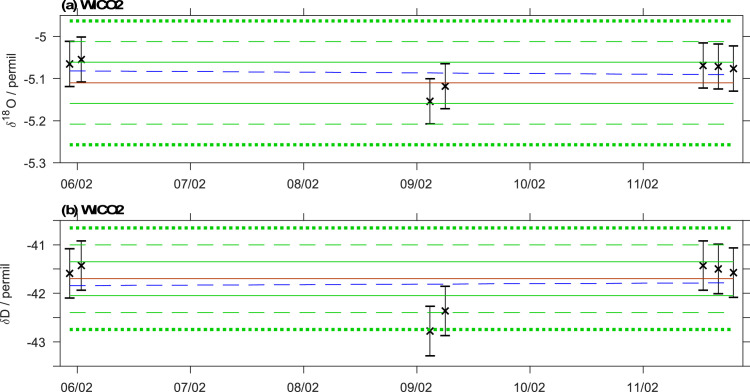
Assessment of the calibration quality from the (a) δ^18^O (‰) and (b) δD (‰) of control standard WICO2 (propagated uncertainty given as bars). The orange solid lines give the assigned values of the standard, and the solid, dashed and dotted lines indicate 1, 2 and 3-σ standard deviations of the long-term reproducibility. Blue dashed line shows a linear fit to the standards after linearity correction.

The overall analytical quality of a run can be assessed from the evaluation of a set of control standards. Control standards are for example included in the measurement sequence of the standards at the beginning of a run. The calibrated drift standards can also serve to assess the quality of a run. In the calibration report, the calibrated values of the control standards are displayed in comparison to their assigned value. Fig. 8 shows an example where WICO2 was used as a control standard. At FARLAB, runs are further inspected and potentially rejected if the majority of the control and drift standards is further than ±2 times the long-term reproducibility of the measurement system from the assigned value (Fig. 8, dashed and dotted green lines).

### Output files and data report

After a click on the *Calibrate!* button in step 5, FLIIMP creates all output files and the calibration report in a new folder within the previously specified output folder (Sec. 3, Fig. 2). Two versions of the calibration report can be created, a user report, and a detailed report (Table 2). The user report will contain only the information that is needed by regular end users, including a table with the calibrated results themselves, a description of the processing steps, a recommendation for acknowledgements, and the calibrated data file with the results for each individual sample. The detailed report contains additional sections that inform about the performance of the measurement system, and the corrections for each sample. For most end users such additional information is either unnecessary, would require extensive description to be useful, or it could be considered non-public information of the laboratory. Therefore, external end users generally receive the simpler user report, whereas the detailed report is archived at the laboratory for traceability and as documentation in case of reprocessing.

**Table 2:** Sections in the FLIIMP processing and calibration reports. Reports are available in a detailed format for internal laboratory use, and in a shortened format for external users.

Section title	Report type	Contents
1. Summary	both	A table of the key parameters and settings applicable to the run
2. Calibrated measurements	both	A table with the calibrated measurements for each sample, two overview figures comparing raw and calibrated data, and links to the output files in csv format.
3. Acknowledgement section for publications	both	A statement that can be used to acknowledge the laboratory of the performed analysis in a publication.
4. Method description for publications	both	A section describing the analytical procedures used for the laboratory analysis for use in publications or similar.
5. Memory correction	detailed	A section describing the memory correction procedures, including a figure presenting the fitting of correction function.
6. Drift correction	detailed	A section describing the linear drift correction.
7. Calibration to VSMOW-SLAP scale	detailed	A section describing the calibration standards, including a table with the calibrated values of the drift standards figure with the time evolution of the calibration standards an drift and control standards in relation to long-term reproducility.
8. Preprocessing and correction details	detailed	A section detailing the excluded samples and injections, a table and a figure showing the impact of mixing ratio–isotop dependency correction, and of the memory correction.
9. Instrument stability	detailed	A section containing a table with the instrument and spectros parameters and error codes, including a figure of the time evolution of several parameters.
Appendix	detailed	Raw data for all vials, including figures that show the raw val each isotope species and for mixing ratio for each injection.

The name of the output folders is created from a combination of the project name, the current run identifier, the processing date, and the report type in the format <project_name>_<run_ID>_<processing_date>_<report_type>, for example 2022–02-HS_run01_20220509_internal. The project name and run identifier should not contain space or file system characters, such as forward and backward slashes, and colons. Within the output folder, the report is created as a HTML document named index.html, to be viewed with any regular browser (Table 3). The HTML report contains links to the result files in csv format, located in the output folder (Table 3). In addition to the calibrated data file and the calibrated data summary, the detailed report contains additional output files for monitoring which samples have been run at what time and position (accounting file), for long-term monitoring of the performance of the measurement system (parameters file, standards file), and for assessing the impact of the corrections and calibration on each measurement (alldata file) (Table 3).

**Table 3:** Output files in comma separated value format created during processing with FLIIMP.

File name	Purpose	Description
<run_ID>_accounting	keeping track of analysed samples for lab management	sample and run identifiers
<run_ID>_alldata	control of corrections and calibration	raw, corrected, and calibrated values for all data fields
<run_ID>_calibrated	final dataset	calibrated values and uncertainty for individual samples
<run_ID>_replicates	averages of replicate samples	calibrated values and uncertainty for replicate samples from the same run
<run_ID>_parameters	quality control of measurement system	run information and fitting parameters
<run_ID>_standards	assessment of long-term reproducibility	calibrated values and uncertainty of drift standards

### Processing of data files with δ^17^O measurements

FLIIMP has been designed throughout as a triple-isotope enabled processing tool. The processing of measurements that include the parameter δ^17^O in addition to δD and δ^18^O require additional user attention [15,17]. In FLIIMP, additional display and processing options become available if δ^17^O values are detected in the input files. In the user interface, the δ^17^O and ^17^O-excess are available as additional display menu options during the sample screening (Step 2). Memory effects for δ^17^O can be corrected during the regular memory assessment (Step 3). Finally, in the calibration settings (Step 5), the user can select whether to include δ^17^O and ^17^O -excess in the output or not using the *Parameter* selector. If δ^17^O output is selected, additional columns are included in the output files for δ^17^O, ^17^O-excess, and their uncertainty. In addition, the format of both δ^18^O and δ^17^O become 4-digit precision, rather than 2-digit, following the recommendations of Schoenemann et al. [15]. In order for δ^17^O analysis to work correctly, the assigned values of δ^17^O for the calibration, drift and control standards need to be included in the csv files defining the calibration standards. While FLIIMP at its present stage enables processing δ^17^O files, users are advised to be aware of additional requirements of this type of analysis, such as sensitivity to drift and large uncertainties from calculation of the ^17^O-excess, and take corresponding measures during sample analysis as recommended in the literature.

## Quality assurance

Due to the consistent treatment of runs, and the output of control parameters of the measurement system, FLIIMP gives direct access to information about the long-term performance of the measurement procedures and specific analysers. In addition, during the development of FLIIMP, care has been taken to test and verify all functionality of the software. A set of test routines allows to produce artificial data sets with defined error properties for drift, memory, or humidity variation, that can then be used to check for correction implementation of new or modified correction routines, and to compare to other calibration methods. Such essential parts of the quality assurance are detailed and discussed below.

### Long-term reproducibility

**Fig. 9:**
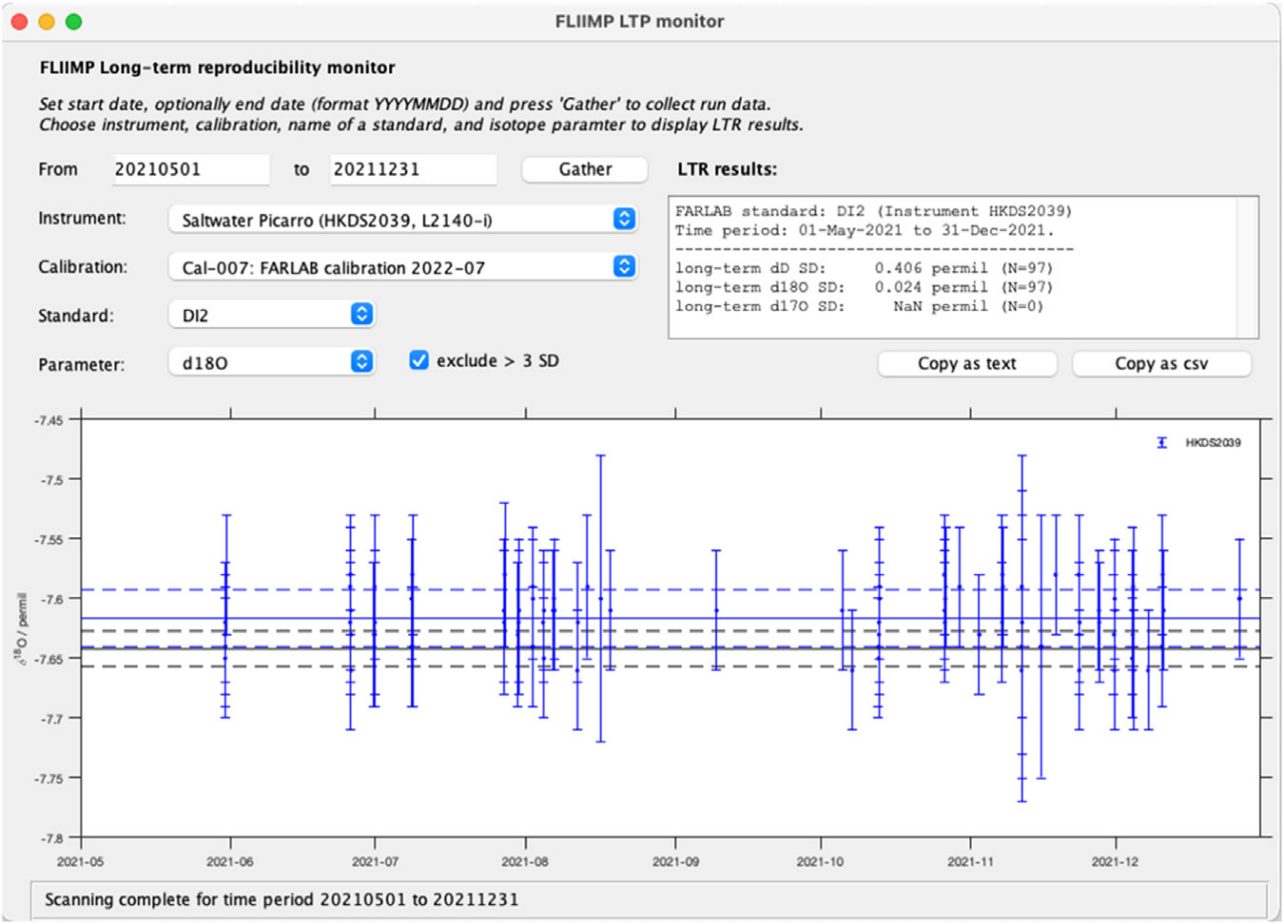
User interface for the assessment of long-term reproducibility within FLIIMP. Shown is an example for 7 months of measurements of drift standard DI2 (within-run 1-σ standard deviation given as error bars) on a Picarro L2140-i analyser with serial number HKDS2038. Here, filtering of runs with a deviation from the assigned value larger than 3 times the long-term reproducibility has been activated.

The long-term reproducibility is an important metric for quality control at a laboratory. Repeated measurements of a control standard over time allows laboratories to quantify their long-term reproducibility. FLIIMP facilitates access to this information by an additional tool that scans through the archived output data files from previous FLIIMP calibrations for a specified time period. The interface to inspect and compute the long-term reproducibility is available from the menu item *Tools, Long-term reproducibility* (Fig. 2). After selecting the menu item, the LTR tool window appears, one can specify the time period and instrument to be analysed (Fig. 9). The assessment results can be copied for further processing into text and spreadsheet applications.

At FARLAB, for example, long-term reproducibility has been quantified from a long-term average of calibrated control/drift standard measurements. During 2016–2020, drift standard DI showed a long-term reproducibility of 0.491 ‰ for δD, and 0.076 ‰ for δ^18^O, estimated from the 1-σ standard deviation of all accepted runs. Runs with large offsets in the control and drift standards (>3 times LTR difference from assigned value), that are archived but have been discarded and repeated, have been excluded from this assessment. Other drift standards result in similar estimates, albeit for shorter time periods. In 2020, drift standard DI2 replaced DI, with a long-term reproducibility of 0.446 ‰ for δD, and 0.052 ‰ for δ^18^O after filtering for runs with a large standard deviation (Fig. 9). To enable inclusion the LTR values in combined uncertainty computation, these need to be specified in the configuration file as parameter longTermReproducibility (Table B.1).

### Comparison to SICalib

We have made a comparison with artificially created input data files processed by both FLIIMP and SICalib (see Sec. 4.4). Thereby, the drift and the memory factors are exactly known, since they have been used in the creation of the artificial data files. Comparisons with these artificial data files with imposed drift show that the memory correction results in differences of the final results that can exceed analytical uncertainty, confirming that the distinction between drift and memory is a main factor for processing. We have also compared the calibrated results between FLIIMP and SICalib for actual runs performed on two different Picarro analysers at the water isotope laboratory at AWI, Germany. When the same actual run files are processed, the root-mean square error amounts to 0.0053 to 0.0227 ‰ for δ^18^O, and 0.032–0.136 ‰ for δD, with a mean error (bias) of −0.0007 to 0.000 ‰ for δ^18^O and 0.016–0.027 ‰ for δD, substantially below typical analytical uncertainties. Since differences in all other processing results appear virtually negligible, we conclude that one can expect equally valid results from the using either of the liquid sample processing tools.

### Processing in batch mode

It may sometimes be necessary to re-process runs that have already been processed interactively with FLIIMP. For example, re-processing may be recommended after recalibration of internal laboratory standards. Other situations that can induce the need to re-process runs with FLIIMP are software changes, such as improved processing or correction methods. The capability to re-process runs in batch mode has also been very useful during the development of FLIIMP to obtain consistent data file formats, allowing to assess the long-term characteristics of the measurement system, and the long-term reproducibility (Sec. 4.1). Batch mode requires a full MATLAB installation with license.

In order to run FLIIMP in batch mode, one or more settings files (*.mat) saved from interactive mode are needed. The setting files are first loaded into a MATLAB variable in a new, separate MATLAB script. Then, the settings variable can be modified as needed, before calling the batch-mode processing routine FLIIMP_batch.m from within the script, using the modified settings variable as an argument. An example for a batch mode script that reprocesses a run while changing from detailed to short reporting is provided in routine FARLAB_batch_run.m in the FLIIMP repository. A description of all elements of the settings structure that can be modified is given in Appendix B.4.

### Test routines for optimised software development

All steps and properties of the FLIIMP software have been quality checked using regular and artificial data files. Artificial data files consist of a text file in the same csv format as obtained from regular analyser output during liquid injection measurements, but with specific characteristics. For example a fixed linear drift in δ-value is imposed across the run. In total, 6 tests have been designed to include drift, memory effects, humidity variation, missing or bad injections, and a combination of several factors (Table 4). The input and output file of the tests are stored in subfolder tests in the FLIIMP source directory.^3^ Test run settings for test 1–6 can either be loaded in the user interface (e.g., test01_run_settings.mat), or users can select instrument HKDS2038 and date 20200101 to 20200106 during processing Step 1, given that the input path points to folder test. Test run files also contain δ^17^O.

**Table 4:** Available artificial test data files for testing the functioning of the FLIIMP software.

Test	Purpose	Imposed modification	Parameter
1	Calibration	constant offset	offset of -12 ‰ for δD and 2 ‰ for δ^18^O and δ^17^O
2	Drift correction	linear drift	drift of 8 ‰ day^−1^ for δD and1 ‰ day^−1^ for δ^18^O and δ^17^O
3	Memory correction	memory effect	memory effect of 1% for δD, δ^18^O, and δ^17^O
4	Mixing ratio dependency correction	mixing ratio variation	random H_2_O mixing ratio variation with a standard deviation of 3000 ppmv
5	Calibration with missing injections	constant offset and missing injections	as Test 1, plus 8 missing injections
6	Realistic use case	all of the above and preconditioning sample	Test 1 to 5 combined, plus a preconditioning sample

**Fig. 10:**
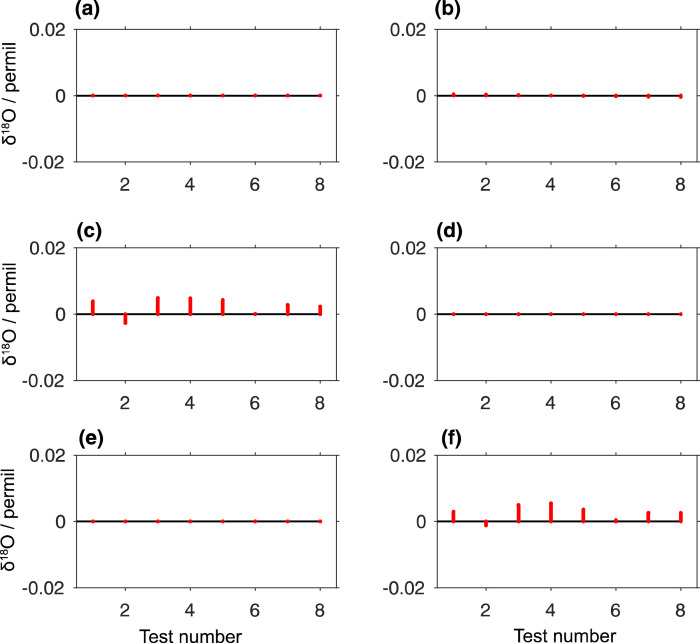
Difference in δ^18^O between calibrated and assigned values in artificial data files with 8 samples according to the FARLAB run setup with prescribed measurement artefacts. (a) Test 1 (constant offset), (b) Test 2 (linear drift), (c) Test 3 (memory effect), (d) Test 4 (Mixing ratio variations), (e) Test 5 (Constant offset and bad injections), (f) Test 6 (Combination of all artefacts and preconditioning sample).

Software tests are common practice in software development to identify software error or other unwanted side effects. The artificial input data files are used to evaluate the correct performance of FLIIMP after, for example, including new or improved functionality. The tests are run using routine batch_FLIIMP_tests.m, which batch processes each test run, then calculates the RMSE of the calibrated values in comparison to assigned values. Tests are passed if the differences in calibrated results are smaller than a specified threshold. Tests of single factors for the current FLIIMP version show close agreement between assigned and calibrated values (Fig. 10, Test 1, 2, 4, 5). Larger deviations are noticeable in the test involving memory correction, and a combination of all factors (Test 3, 6). This larger difference can be mainly ascribed to the previously mentioned difficulties to separate instrument drift and memory effects during post-processing. New test input files can be created using the routine create_FLIIMP_tests.m.

## Final remarks

FLIIMP is a flexible, new analytical tool for processing liquid sample measurements for stable water isotopes from commercially available laser spectrometers, that runs on different operating systems. FLIIMP provides calibrated results that can be considered equal to other existing solutions, while proving several innovations that reduce time efforts during processing, make operations less error-prone, and support the traceability of results by consistent documentation.

The availability of a GUI allows for user guidance and interactive adjustment of parameters, for example during sample screening and memory correction, and the quality assessment of individual runs, and for the analytical system. Runs may be reprocessed in batch mode if needed, to obtain consistent long-term monitoring of a measurement system, including long-term reproducibility. Standardised reports in HTML format contain the information for either customers or laboratory operators. Data files are made available in csv format for simple use in either spreadsheet applications or data analysis software. FLIIMP thereby contributes to overall more standardised processing in laboratory workflows, and supports principles of good laboratory practice, such as traceability.

While FLIIMP can be run without MATLAB license using MathWork's runtime environment, an important current limitation of FLIIMP is that further development requires a costly software license. While on the longer term a transition to the license-free python language can be achieved, the stand-alone compiled version of FLIIMP are available as a first-order remedy. Another important limitation is that FLIIMP currently is only able to read input files from Picarro-brand analysers. With FLIIMP being available in a publicly accessible repository, the community may actively contribute with improved processing methods to the future development of FLIIMP.

Processing of measurement files and artificial input files show that correcting for memory across injections and across samples, and the separation between drift and memory effects, remain a major challenge for liquid water analysis for the instruments used here. Flexible post-processing tools, such as FLIIMP, are a valuable asset in finding run setups and other developments that further improve correcting methods and analytical procedures, thereby supporting reliable scientific interpretations of stable water isotope measurements from different laboratories.

## Ethics statements

Not relevant for this work.

## CRediT authorship contribution statement

**Harald Sodemann:** Conceptualization, Software, Writing – original draft. **Pål Tore Mørkved:** Methodology, Validation, Writing – review & editing. **Sonja Wahl:** Writing – review & editing.

## Declaration of Competing Interest

The authors declare that they have no known competing financial interests or personal relationships that could have appeared to influence the work reported in this paper.

## Figures and Tables

**Fig. A.1:**
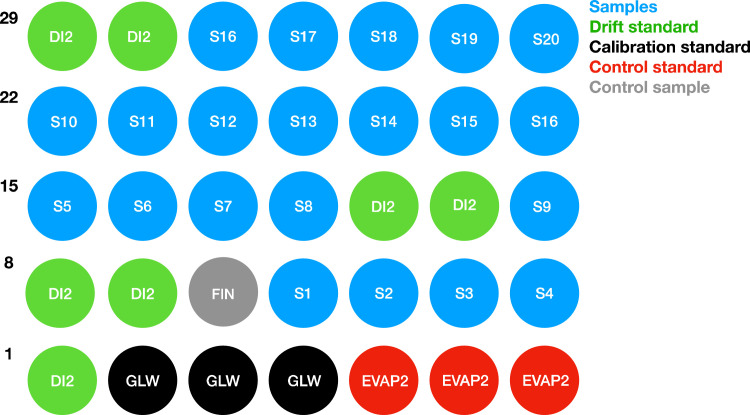
Current run set-up for the liquid sample analysis for meteoric water (precipitation), run-off, sea water, and ground water at FARLAB. S1-S20 are samples (blue), BERM is a control sample (grey), GLW and EVAP2 are calibration standards (black), FIN is a control standard (red), and DI2 is the drift standard (green).

**Table B.1:** Description of parameter values for the configuration of FLIIMP contained in file *FLIIMP_config.csv*.

Parameter	Type	Default value	Description
installdir	s	/Users/hso039/Dropbox/FARLAB/matlab/FLIIMP/src	installation directory of source code
defaultInputPath	s	/Volumes/farlab	default data file input base path
defaultOutputPath	s	/Volumes/farlab	default report output base path
archivePattern	s	%s/Projects/%d/	default archive path pattern, filled by input path (%s) and year (%d)
inputPattern	s	%s/Instruments/%s/IsotopeData/	default input path pattern, filled by input path (%s) and instrument (%s)
logofile	s	FARLAB_logo_circle.png	name of logo file for reports, located in installdir
version	s	V2.0, (c) 2018-2023 Harald Sodemann (harald.sodemann@uib.no)	version string
labname	s	'FARLAB'	laboratory name
devices	a	{'HKDS2038′;'HKDS2039’;'HIDS2254’;'HIDS2380′}	instrument serial identifiers
fileType	s	_IsoWater_	input file name component <device><fileType><date and time>.csv
deviceNames	s	{'Freshwater Picarro (HKDS2038, L2140-i)';'Saltwater Picarro (HKDS2039, L2140-i)';'Vapour Picarro (HIDS2254, L2130-i)';'ISLAS Picarro (HIDS2380, L2130-i)'}	instrument descriptive names
longTermPrecision	a	'[0.049 0.349 0.049 NaN NaN]’	long-term reproducibility, obtained from 2019-2020 measurements of drift standard DI at FARLAB for d18O, dD, d17O, dxs, e17
reference	s	‘<a href=“http://www.uib.no/en/BGF/FARLAB”>FARLAB</a>, University of Bergen’	reference to laboratory for report
acknowledgement	a	'Handling and processing of liquid stable isotope samples was done at the Norwegian National Infrastructure project FARLAB (Facility for advanced isotopic research and monitoring of weather, climate, and biogeochemical cycling, Project Nr. 245907) at the University of Bergen, Norway.\\n'	acknowledgement statement for report
memory_limits	s	[1.5,12,1.5,0,0]	memory correction limits for minimal inbetween-sample difference to apply correction for d18O, dD, d17O, dxs, e17
H2O_min	s	15000.0	quality flag limit H2O minimum
H2O_max	a	25000	quality flag limit H2O maximum
H2O_SD	n	200.0	quality flag limit H2O standard deviation
H2O_inj_SD	n	500.0	quality flag limit H2O per-injection standard deviation
dD_SD	n	0.5	quality flag limit $\delta$D SD
d18O_SD	n	0.15	quality flag limit $\delta^{18}$O SD
T_DAS_SD	n	0.15	quality flag limit DAS temperature SD
warning	n	0	show all warnings (0=off, 1=on)

**Table B.2:** Description of parameter values for a specific run to be processed with FLIIMP. Parameter values are modified by the GUI of FLIIMP, but can also be modified programmatically when running FLIIMP in batch mode.

Parameter	Variable type (range)	Description
averageFirstInjections	integer	number of last injections to average (first sample)
averageInjections	integer	number of last injections to average (all other samples)
calibrationRun	integer (1…nr. of calibrations)	index of calibration set
calibrationType	integer (1)	type of calibration (two-point=1)
contact	string	customer contact email
customer	string	customer name
endDate	string (YYYYMMDD_HHMMSS)	end date of input file search
error	integer	error flag
excludeInj	cell array of integer arrays	injections to exclude for each sample
ignoreInjectionsCal	integer array (0/1)	array for vials to include in calibration
ignoreInjections	integer array (0/1)	array for vials to include in memory correction
ignoreVialsCal	double array	array with indices of vials to include in calibration
ignoreVials	double array	array with indices of vials to include in memory correction
includeE17	integer (0/1)	include 17-O and 17-O excess
inputFiles	cell array of strings	input files for manual file selection
inputPath	string	input path
instrument	string	instrument identifer
mem	struct	memory correction parameters
operator	string	instrument operator name
operatorContact	string	instrument operator contact
outputPath	string	path for output files
project	string	project identifier
rename	struct of cell arrays	information for renaming of samples
reportDate	integer (matlab datenum)	date of reporting
runID	string	run identifier
standardNames	cell array of strings	names of calibration standards
standardsDrift	cell array of strings	names of drift and control standards
startDate	string (YYYYMMDD_HHMMSS)	start date of input file search
useDriftCorrection	integer (0/1)	use drift correction
useHumidityCorrection	integer (0/1)	use humidity dependency correction
useMemoryCorrection	integer (0/1)	use memory correction
writeReport	integer (0/1/2)	write long or short report, or both

## Appendix A Analytical workflow

FLIIMP is easily embedded in the typical analytical workflow of a laboratory. We provide here the example of the steps involved at FARLAB from sample receipt to reporting. These steps may differ at other laboratories, and partly be provided from other laboratory management solutions. Importantly, FLIIMP is not tied to a specific run setup, and works with any run setup with 2 point calibration, independently of the number of injections and sample and standard repetitions.

### Appendix A.1 Run setup example from FARLAB

Below we provide an example for the analytical setup for liquid water isotope analysis currently in use at FARLAB. For the setup of a run on a laser spectrometer, samples are filtered if needed, and transferred to 2 ml GC-vials according to established routines [9]. In the autosampler tray, runs are then set up with 4 standards for calibration, control, and drift for quantification of short-term and long-term reproducibility. The first vial is a pre-conditioning sample, to allow for memory correction of the first standard (Fig. A.1). Thereafter, triplicates of the calibration standards are injected, followed by a duplicate of the drift, and the control standard measured with the same number of injections as the following samples. After every 5–8 samples, a set of two drift standard vials is measured, treated as a regular sample. During processing, the first vial of the drift is rejected as it is still potentially affected by memory effects. Calibration standards are typically injected 12–16 times to enable memory correction, while samples are typically injected 10 times. At FARLAB, we have implemented a wet flush method for the first two injections, where the vaporiser and analyser inlet are flushed briefly with a vapour stream at double the typical humidity. This humidity peak reduces memory from previous samples to some extent, and does not appear in the output files. The effect can be compared with 2 regular injections, but with about 50% shorter duration. The typical duration of a run with 20–30 samples in this setup is about 2–3 days.

### Appendix A.2 Operating checklist

The operating checklist below assumes that FLIIMP has been installed as described in Sec. 2, and works as expected.

Prerequisites:

• Mount network drives that contain original data files
• Start MATLAB and change current directory to the source directory of FLIIMP, then run FLIIMP.m
• Alternatively, start the compiled FLIIMP version.

Processing Step 1: Paths and files

• Select input path. This is the folder where output files from the analysers are located.
• Select output path. Normally, this is a folder for a specific project. Example: YYYYNNNUU_description.
• Select the analyser from the drop-down menu.
• Select the start date/time of the run. If needed also specify (parts of) the start time. Format: YYYYMMDD_HHMMSS
• If the entire run is contained in one file, the end date may be empty. To process a time range, also add an end date, and all files found for days in between will be included.
• Alternatively, select specific file(s) from any location using the file selector.

Processing Step 2: Pre-processing and selection

• Inspect log output for advice on aspects to check.
• Check for measurements with low measurement frequency.
• Click on each sample to inspect for injection problems
• Exclude poor injections by manually adding injection number or clicking above/below injection in error bar plots
• Exclude an entire sample by button ’Exclude from calibration’, for example duplicates and pre-conditioning samples.

Processing Step 3: Memory correction

• Open memory correction panel by the ‘Memory correction’ button
• Enter the minimum number of injections required, or alternatively, enter the names of the standards to be included.
• Samples that should not be included but fall under these selection criteria can be excluded using ‘Exclude from memory correction’ checkmark in the pre-processing window (Step 2).
• Press autofit to find the best fit and adjust fitting results as needed
• Repeat for all isotope species
• Decide whether to use memory correction or not using the checkbox
• Press ‘Apply’ to accept memory correction
• Inspect green markers for standards and for all vials to evaluate the quality of the memory correction, iterate as needed

Processing Step 4: Renaming samples

• Check for mixed up, missed or mistyped sample names and sample IDs
• Change individual samples as needed
• Create a missing laboratory ID as needed
• Specify standard names to jump over these vials during automatic lab ID assigment
• Processing Step 5: Run information and sample calibration
• Specify project name and run ID
• Specify name and email for operator and customer
• Choose calibration set
• Specify calibration and drift standards, with standard names separated by spaces
• Specify averaging of injections for first and all other samples, use −1 to include all
• Preview calibration and drift using the corresponding buttons
• Select the report type for internal and/or external use. External customers usually receive an abbreviated version of the report that does not disclose lab internal instrument data.
• Save settings any time underway, but at the latest now
• Press calibrate and watch for report being generated
• Processing Step 6: Check results
• Open report file index.html in a browser and inspect the results, in particular the memory correction, drift correction, calibration, and uncertainty.
• Return to FLIIMP to make adjustments if needed

## Appendix B Configuration of FLIIMP

While FLIIMP can be tested without modifications using the provided sample input file, laboratories eventually will have to adapt FLIIMP to their specific environment. All configuration settings are collected in csv file FLIIMP_config.csv located in the source directory (Table B.1). In order to adapt FLIIMP to a new laboratory, the settings within this file need to be adapted. Some settings require adjustment in all cases, such as the laboratory and instrument names. Other settings are recommended to be adjusted to include the correct method description and acknowledgement sections. Optionally, one can specify the correction functions of the mixing ratio–isotope ratio dependency. After these initial modifications, FLIIMP can be run interactively or in batch mode in a new laboratory. New standards can be added as described in the next section. More complex changes involve modifications in other parts of the program code and are described further below.

### Appendix B.1 Specifying new standards

Laboratories will need to specify their laboratory standards during FLIIMP configuration. Available standards are specified as csv files in the subfolder standards. A new calibration set is most easily created by copying an existing calibration set to a new name, specifying a number at the start of the file name followed by a dash (e.g., 004-lab-standards-2023.csv). Each row in the csv file corresponds to a standard, and each column contain the assigned value and uncertainties for the three isotope species (dD, dD_u, d18O, d18O_u, d17O, d17O_u for δ_D_, δ^18^O, and δ^17^O). A description string allows to identify the used calibration in the data report. During the build-up of the user interface, the calibration sets in the standards folder are automatically identified and made available from the respective pull-down menu (e.g., Fig. 7, Calibration standards).

### Appendix B.2 Input file format and pathname templates for file input

FLIIMP currently parses analyser input files in expectation of a specific text file format for Picarro-brand analysers, e.g., HKDS2039_IsoWater_20220101_102000.csv. The order of data columns can vary, and if not all fields are present in a file, a warning message is displayed, and FLIIMP attempts to construct missing information if possible. The file input takes place in routine FLIIMP_read_liquid_injections.m, and input functionality for other analysers brands may be added here.

At FARLAB, the liquid injection data files from all analysers are archived in a file structure with a base path contained in variable inputPath (e.g., /Volumes/farlab/. Within the base path location, FLIIMP expects a sub-folder Instruments. Therein, subfolders named as the instrument identifiers are expected (e.g., HKDS2039, each containing a sub-folder IsotopeData, which then finally contains all liquid injection data files. The complete search path to the data files is thus composed by joining several parameters in the form <inputPath>/Instruments/<instrument>/IsotopeData/. When adapting FLIIMP to a new lab, one can either choose to reproduce the file structure expected by FLIIMP, or modify the parameter inputPattern in FLIIMP_settings.csv as needed (Sec. 2).

### Appendix B.3 Specifying mixing ratio – isotope ratio correction functions

Measurements at varying mixing ratios are affected by changes in base line during spectroscopic analysis (see Sec. 3.2.2). In FLIIMP, corrections to the mixing ratio-isotope ratio dependency are implemented in file FLIIMP_wconc_corr.m. In the routine, a case statement selects from which analyser the data originate. Corrections are either specified in a linear relation between corrected and raw signal or in a more complex correction function that also includes the δ-value (Sec. 3.2.2). The corrected δ-values are then assigned to a new variable ending in _corr. If no correction functions are available, the corrected and uncorrected values are identical, and a warning is displayed in the log. This behaviour can be deactivated by unchecking the humidity correction check box during Step 5, or permanently by uncommenting the statement use = 0 in routine FLIIMP_wconc_corr.m.

### Appendix B.4 Structure of the settings variable

For running FLIIMP in batch mode, the common procedure is to read in saved settings files from a previous interactive FLIIMP session. The settings file is in *.mat format, and can be read in before modification of different elements. The contents of the settings variable (and file) are described in Table B.2, including the data types and formatting requirements.

## Footnotes

1 https://git.app.uib.no/GFI-public/fliimp.

2 fliimp-master/tests/Instruments/HKDS2038/IsotopeData/HKDS2038_IsoWater_20220205_152236.csv

3 fliimp-master/tests/Instruments/HKDS2038/IsotopeData/
